# Assessment of the N-PCR Assay in Diagnosis of Pleural Tuberculosis: Detection of *M.tuberculosis* in Pleural Fluid and Sputum Collected in Tandem

**DOI:** 10.1371/journal.pone.0010220

**Published:** 2010-04-19

**Authors:** Parameet Kumar, Manas K. Sen, Devendra S. Chauhan, Vishwa M. Katoch, Sarman Singh, Hanumanthappa K. Prasad

**Affiliations:** 1 Department of Biotechnology, All India Institute of Medical Sciences, New Delhi, India; 2 Department of Pulmonary Critical Care and Sleep Medicine, Vardhman Mahavir Medical College (VMMC) and Safdarjung Hospital, New Delhi, India; 3 National JALMA Institute of Leprosy and Other Mycobacterial Diseases, Tajganj, Agra, India; 4 Department of Health Research (Ministry of Health & Family Welfare) and Indian Council of Medial Research, Ramalingaswami Bhawan, Ansari Nagar, New Delhi, India; 5 Division of Clinical Microbiology, Department of Laboratory Medicine, All India Institute of Medical Sciences, New Delhi, India; St. Petersburg Pasteur Institute, Russian Federation

## Abstract

**Background:**

The nonspecific clinical presentation and paucibacillary nature of tuberculous pleuritis remains a challenge for diagnosis. Diagnosis of tuberculous pleural effusion depends on the demonstration of the presence of tubercle bacilli in the sputum, pleural fluid, or pleural biopsy specimen, or demonstration of granuloma in pleura by histological examination. We examined the clinical utility of the diagnosis of pleural tuberculosis using the in house N-PCR assay, AFB smear microscopy and culture. Besides pleural fluid the inclusion of sputum in the efficacy of diagnosis of pleural tuberculosis was scrutinized.

**Methodology/Principal Findings:**

Pleural fluid and sputum samples of 58 tuberculous and 42 non-tuberculous pleural effusion patients were processed for AFB smear microscopy, culture and the N-PCR assay. Mycobacteria were detected exclusively in tuberculous pleural effusion samples. None of the non-tuberculous pleural effusion samples were positive for mycobacteria. Comparative analysis showed that the N-PCR assay had the highest sensitivity. Inclusion of sputum along with pleural fluid increased N-PCR sensitivity from 51.7 to 70.6% (p<0.0001).This improved sensitivity was reflected in AFB smear microscopy and isolation by culture. The sensitivity enhanced on inclusion of sputum from 3.4 (p = 0.50) to 10.3% (p = 0.038) for AFB smear microscopy and for isolation of mycobacteria from 10.3(p = 0.03) to 22.4% (p = 0.0005). Thirteen isolates were obtained from 58 pleural tuberculosis patients. Eleven mycobacterial isolates were identified as *M.tuberculosis* and two as *M.fortuitum* and *M.chelonae*. Complete concordance was seen between the biochemical identification of isolates and the N-PCR identification of mycobacterial species prior to isolation.

**Conclusions/Significance:**

To the best of our knowledge this is the first PCR based report on utility of sputum for diagnosis of pleural tuberculosis. The present study demonstrates that a combination of pleural fluid with sputum sample and N-PCR improved the diagnosis of pleural tuberculosis.

## Introduction

Pleural Tuberculosis is a common manifestation of extra pulmonary tuberculosis and is a frequent cause of pleural effusion [1]–[2]. Tuberculous pleural effusion (TPE) occurs in up to 30% of tuberculosis patients [3]. Its occurrence in developing countries has increased following the HIV pandemic [4].

Rapid diagnosis and administration of prompt anti-tuberculous treatment of pleural tuberculosis is needed to reverse the morbidity due to tuberculosis (TB). Owing to the paucibacillary nature of the pleural fluid, the diagnosis of tuberculous pleuritis is a challenge [5]. Hence in addition to collection of pleural fluid in suspected cases of Tuberculous pleural effusion, it has been recommended that sputum be included for laboratory investigation as an additional sample, [6]–[7]. To improve the detection of mycobacterial pathogens in paucibacillary samples inclusion of sensitive probes such as molecular beacons would be an added advantage, [8]. The definitive diagnosis of pleural tuberculosis is currently made by demonstrating the presence of tubercle bacilli in specimens such as sputum/pleural fluid and/pleural biopsies, or by histological examination of pleural tissue for granulomas [4]. As these methods are disadvantaged by the sensitivity and time required for detection and identification of mycobacterial pathogens in clinical samples, in the present study, we examined the potential of DNA amplification techniques to rapidly detect *Mycobacterium tuberculosis*/*Mycobacterium bovis* in pleural fluid. Besides pleural fluid the clinical utility of inclusion of sputum for diagnosis of pleural tuberculosis was also examined. N-PCR targeting the *hupB* gene (*Rv2986c*) was used to detect *Mycobacterium tuberculosis* (*Mtb*) and *Mycobacterium bovis* (*Mbo*) in pleural fluid and sputum samples collected in tandem from pleural effusion patients. The utility of the assay in the detection of mycobacterial pathogens namely *Mtb* and *Mbo* in clinical samples has been previously reported [9]–[10]. The results obtained using the N-PCR assay was compared with Acid Fast Bacilli (AFB) microscopy and isolation and identification of AFB by culture present in pleural fluid and sputum derived from pleural effusion patients.

## Materials and Methods

### Patient's selection & study design

#### Ethics Statement:

The study, information sheet and consent form used was approved by the institutional ethical committees of the All India Institute of Medical Sciences, New Delhi & Vardhaman Mahavir Medical College & Safdarjung Hospital, New Delhi-110029. India. Informed written consent from all participants involved in our study has been obtained.

Over a 4 year period, 100 pleural effusion patients with no history of anti-tuberculosis treatment registered in the Department of Respiratory Medicine, Safdarjung Hospital, New Delhi were included in the study. Pleural fluid was aseptically aspirated under sterile conditions in the minor procedure room facility and transported to the laboratory for investigation. The clinical diagnosis was not available during the laboratory investigation. The diagnosis was available during data analysis.

All the patients included in the study were patients with pleural effusion and did not have lung parenchymal involvement. The clinical criteria as described by Light (2001) [11] were adopted for the diagnosis of pleural tuberculosis. The definitive/confirmed criterion for tuberculous pleural effusion was the demonstration of AFB in pleural fluid and or sputum by microscopy or culture. Suggestive/probable criteria included:(1) patients with clinical history of fever, pleuritic chest pain, cough, breathlessness, and chest radiography for evidence of pleural effusion; (2) cytological examination of the pleural fluid for predominance of lymphocytes, paucity of mesothelial cells;(3) biochemical estimation for protein content (>3 gm per dl) and pleural fluid: serum protein ratio (>0.5); and (4) response of patients to anti-tuberculous treatment. Tuberculous pleural effusion was diagnosed if the definitive criterion or all of the suggestive criteria were met. Based on these criteria 58 patients were classified as patients with tuberculous pleural effusion. Sputum as well as pleural fluid was collected from all of them and investigated, [6]–[7].

Criteria used for malignant pleural effusion (controls) were: (i) Clinical history suggestive of rapidly refilling pleural effusion; with or without focal malignant lesion elsewhere in the body. (ii) Pleural fluid being exudative, usually hemorrhagic and (iii) on cytological examination positive for malignant cells. Based on these criteria 42 patients were classified as non-tuberculous pleural effusion patients (N-TPE). In twelve of these patients sputum was collected in addition to the pleural fluid.

### Sample Processing

#### For isolation of mycobacteria:

All the clinical samples were processed in Biosafty level 3 (BSL-3) facility. After collection, samples were transported at 4–8°C and held at this temperature in the facility till they were processed for isolation & DNA extraction. All samples were processed with in 24 hours of collection with freshly prepared reagents. Only 2–3 clinical samples were processed at a time to minimize sample cross contamination by trained personnel.

#### Pleural fluid:

100 pleural fluid samples were processed by NALC-NaOH method [12]. In brief, pleural fluid was centrifuged at 4,500 g for 15 min. The pellet was treated with an equal volume of *N*-acetyl-L-cysteine- NaOH (NALC-NaOH; NaOH final concentration, 2%) for 15 min at room temperature and neutralized with sterile phosphate buffer (0.067 M, pH 6.8). After centrifugation at 4,500 g for 15 min, the pellet was resuspended in 1 ml of sterile distilled water [13]. The suspension was divided into two parts. One part was used for AFB smear microscopy and as inoculums for isolation of AFB by culture on Lowenstein Jensen media & 7H9 or in MGIT tubes (BD BACTEC MGIT 960 system). The second part was used for DNA extraction. Sputum collected from 70 patients was similarly processed.

#### Ziehl-Neelsen smear of pleural fluid & sputum:

Ziehl-Neelsen acid fast staining was used to confirm the presence of AFB.

#### Pleural fluid and sputum culture:

As described processed clinical specimens (Pleural fluid & sputum) obtained from 69 pleural effusion patients were inoculated on LJ slants & 7H9 liquid media and were incubated at 37°C for 8 weeks. LJ slants were inspected twice a week for visible colonies, and were screened for AFB by ZN staining. The liquid media were examined at identical time points for growth. Tubes showing visible growth were screened for AFB by ZN staining. Positive liquid cultures were sub-cultured on to LJ media for isolation of mycobacteria. Following the access to the automated system concentrated clinical specimens (0.5 ml) obtained from 31 pleural effusion patients were inoculated in the MGIT media alone containing PANTA antibiotics mixture as per manufacturers' recommendation and monitored continuously in MGIT 960 system (BD BACTEC™ MGIT™ 960 system for mycobacteria testing, USA).

#### Identification of mycobacteria isolated from pleural fluid and sputum

Species level identification of the isolates was done by standard biochemical tests (niacin production, nitrate reduction, catalase and aryl sulfatase activity, Tween hydrolysis, thiopen-2-carboxylic acid hydrazide sensitivity, etc.) as recommended by the Centers for Disease Control and Prevention (CDC), Atlanta, Ga., with appropriate controls [14]. For this part of the study mycobacterial colonies derived from LJ media were used.

### Extraction of template DNA

#### Positive Control template DNA:

*Mtb* H37Rv & *Mbo* AN5 colonies suspended in 100 µl of 0.1% triton X-100 was boiled in a dry bath (90°C for 40 mins.), centrifuged (10,000 g, for 10 min.) and the supernate was aliquoted & stored at −20°C. The supernatant was used as target DNA in the PCR assay. *Mtb* H37Rv & *Mbo* AN5 strains were obtained from National JALMA Institute for leprosy & other mycobacterial Diseases (ICMR), Agra, India. Besides *Mtb* cultures *Mycobacterium tuberculosis* H37Rv DNA obtained from “TB research Materials and Vaccine Testing” was also used as a control.

Template DNA from clinical samples:. The 2^nd^ portion of the suspension obtained from the processed clinical samples was centrifuged. The pellet was suspended in 100 µl of 0.1% triton X-100 & boiled in a dry bath, (90°C for 40 mins.). The suspension was centrifuged and processed as described for DNA extraction for positive control template DNA.

#### Nested PCR for *hupB* DNA target (International patent application no. PCT/IN03/00302):

The details of the assay have been described previously [10]. The primers **N** (5′-GAGGGTTGGGATGAACAAAGCAG-3′) and **S** (5′-TATCCGTGTGTCTTGACCTATTTG-3′) were used to amplify *hupB* gene. The amplified **N-S** PCR product was used as a template for the N-PCR. The primer pair for the N-PCR **F** (5′-CCAAGAAGGCGACAAAGG-3′) and **R** (5′-GACAGCTTTCTTGGCGGG-3′) was used. The N-PCR products were electrophoresed on 10% polyacrylamide gel and stained with ethidium bromide. The amplicon size for *Mtb* and *Mbo* was ∼116 bp and 89 bp respectively [15]. For the N-PCR assay, the following controls were routinely included: negative control (with out template DNA) was incorporated in the assay to rule out the occurrence of false-positives. Spiked controls were included to rule out the occurrence of false-negative results in the study due to the presence of PCR inhibitors in DNA extracts of samples. Beside these two controls positive control with target DNA was included in each assay, [10].

#### Statistical Analysis:

All statistical analyses were done using STATA software, version 9.2 (StataCorp, College Station, TX). For all analyses, *p* values less than 0.05 were considered to indicate statistical significance.

## Results

The gender and age distribution of 100 pleural effusion patients collected were as follows: 35 females and 65 males with a mean age of 42±8.8 and 40±4.3 respectively. Based on clinical criteria described 58 patients were categorized as TPE and the remaining as N-TPE. AFB was detected by smear microscopy and or by culture in 16 samples (sputum &/pleural fluid) derived from TPE patients. These individuals were considered to be definitive/confirmed cases of pleural TB (16/58, 27.5%). The remaining 42 in the TPE category were considered as probable cases of pleural TB, (42/58, 72.4%). The N-PCR results were compared with: (i) clinical diagnosis; (ii) improvement in the detection of mycobacteria in sputum samples besides pleural fluid; (iii) patients segregated as AFB positive (confirmed TB pleurisy) Vs AFB negative (probable TB pleurisy); and (iv) biochemical characterization of AFB isolates.

### Detection of mycobacteria in pleural fluid samples

N-PCR, culture and AFB smear microscopy were compared and have been shown in Table 1. In 31 samples, mycobacteria were detected either by N-PCR/culture/AFB smear microscopy. These 31 samples were derived from the 58 clinically diagnosed tuberculous pleural effusion patients (TPE). In contrast, no mycobacteria were detected in the 42 samples obtained from non-tuberculous pleural effusion patients (N-TPE, controls).

**Table 1:** Comparative analysis of 100 Pleural fluid samples with N-PCR, Culture and AFB with clinical diagnosis.

Method		Clinical diagnosis		Sensitivity (%)	Specificity & PPV (%)	NPV (%)	p value
		Patients (58)	Controls (42)				
**N-PCR**	Positive	30	0	51.7	100	60	p<0.0001
	Negative	28	42				
**Culture**	Positive	6	0	10.3	100	44.6	p = 0.038
	Negative	52	42				
**AFB**	Positive	2	0	3.4	100	42.8	p = 0.50
	Negative	56	42				

**NOTE**. Clinical diagnosis, Categorization of patients and controls as describe in methods; PPV, positive predictive value; NPV, negative predictive value; Sensitivity, TP/(TP+ FN) x 100, where T is true, F is false, P is positive, and N is negative; Specificity, TN/(TN+ FP) x 100; N-PCR, 116 bp amplicon generated by Nested-PCR assay specific for *Mycobacterium tuberculosis*; Culture, AFB Growth detected on LJ, 7H9 and/MGIT; AFB, acid fast bacilli.

By N-PCR assay 51.7% (30/58) of the pleural fluid samples derived from TPE were positive for *Mtb* compared to 10.3% (6/58) by culture & 3.4% (2/58) by AFB smear microscopy. In the pleural fluid sample negative by the N-PCR assay, the AFB isolated from the sample was subsequently identified as *Mycobacterium fortuitum* (Sample No. SPF-8). The six isolates were obtained, two each from LJ, 7H9 & MGIT tubes respectively. All the assays showed identical specificity & positive predictive value (PPV) but differed in their sensitivity & negative predictive value (NPV). N-PCR assay showed the highest sensitivity (51.7%) compared to culture (10.3%) and AFB smear microscopy (3.4%, Table 1).

### Detection of mycobacteria in sputum samples collected from pleural effusion patients

Based on criteria described in methods the distribution of the 70 sputum samples collected from pleural effusion patients is shown in Table 2. Fifty-eight sputum samples were from patients with tuberculous pleural effusion and 12 were with non-tuberculous pleural effusion. Identical methods were used for detecting *Mtb* in these sputum samples as described earlier. Of the 70 samples analyzed, in 33 samples *Mycobacteria* was detected either by N-PCR/Culture/AFB smear microscopy. All the 33 positive samples were derived from TPE. The twelve samples collected from N-TPE were negative.

**Table 2:** Comparative analysis of 70 sputum samples with N-PCR, Culture and AFB with clinical diagnosis.

Method		Sputum from tuberculous and non tuberculous patients		Sensitivity	Specificity & PPV (%)	NPV (%)	p value
		Patients (Tuberculous) (58)	Controls (Non- tuberculous) (12)				
**N-PCR**	Positive	31	0	53.4	100	30.0	0.0007
	Negative	27	12				
**Culture**	Positive	7	0	12.1	100	19.0	0.34
	Negative	51	12				
**AFB**	Positive	4	0	6.9	100	18.1	1.0
	Negative	54	12				

**NOTE**. Clinical diagnosis, Categorization of patients and controls as describe in methods; PPV, positive predictive value; NPV, negative predictive value; Sensitivity, TP/(TP+ FN) x 100, where T is true, F is false, P is positive, and N is negative; Specificity, TN/(TN+ FP) x 100; N-PCR, 116 bp amplicon generated by Nested-PCR assay specific for *Mycobacterium tuberculosis*; Culture, AFB Growth detected on LJ, 7H9 and/MGIT; AFB, acid fast bacilli.

Using the N-PCR assay, 53.4% (31/58) of the sputum samples were positive for mycobacteria compared to 12.1% (7/58) by culture and 6.9% (4/58) by AFB smear microscopy. The seven isolates were obtained, two each from LJ, 7H9 & three from MGIT tubes respectively. Of the 33 samples in which *mycobacteria* were detected, two of the samples were negative by the N-PCR assay. In one sample, the AFB isolated was identified as *Mycobacterium chelonae* (Sample No. SPF-80).The second sample was AFB positive but culture and N-PCR negative (Sample No. SPF-18). As seen earlier, the three methods used for detection of mycobacteria in the sputum samples showed similar range of specificity, PPV and sensitivity. All three methods showed 100% specificity & PPV and their sensitivity was 53.4% by the N-PCR assay, 12.1% by culture and 6.9% by AFB smear microscopy.

### Comparison of N-PCR results with detection of AFB & its isolation by culture in pleural fluid & sputum samples

AFB detection in smears and isolation of mycobacteria enhanced, on inclusion of sputum samples besides pleural fluid from clinically diagnosed cases of TPE, (Table 3). In case of culture the isolation of mycobacteria was limited to 6 (10.3%) pleural fluid samples. However inclusion of sputum samples collected in tandem from these TPE patients, mycobacteria were isolated from an additional 7 sputum samples. No mycobacteria were isolated from the pleural fluid samples of these 7 patients, thereby improving the detection of mycobacteria with the inclusion of sputum samples in this category of patients from 6 to 13 (22.4%, p = 0.0005). In case of AFB smear microscopy the number of positive samples increased from 2 (3.4%) to 6 (10.3%, p = 0.038, Table 3), with the detection of AFB in 4 sputum samples, whereas the tandem pleural fluid samples of these patients were AFB negative. Similarly, improved detection of mycobacteria by the N-PCR assay in clinically diagnosed patients of TPE increased on inclusion of sputum samples to that observed by AFB smear microscopy and isolation by culture. By N-PCR in 30 pleural fluid samples *Mtb* was detected. On inclusion of sputum for detection of *Mtb* from these patients, *Mtb* was detected in 11 sputum samples; the concomitant N-PCR results of the pleural fluids derived from these 11 patients were negative by N-PCR. The sensitivity of the detection of *Mtb* by N-PCR assay on inclusion of sputum along with pleural fluid, in TPE patients enhanced from 30 (51.7%, Table 1, 3) to 41 patients (70.6%, p<0.0001, Table 3). Comparing the 3 methods for detection of mycobacteria in the clinical samples, the highest sensitivity was seen in case of N-PCR (70.6%), followed by culture (22.4%) and the least by AFB smear microscopy, (10.3%).

**Table 3:** Detection & distribution of mycobacterial pathogens in sputum & pleural fluid samples collected in tandem from 58 clinically suspected tuberculous pleural effusion patients by N-PCR, Culture & AFB smear microscopy.

Total No. of Samples (58)	Positive for N-PCR	Culture	AFB
Both sputum & Pleural Fluid Positive	34.5% (20/58)	0	0
Sputum alone Positive	19.0% (11/58)	12.1% (7/58) p = 0.3	6.9% (4/58) p = 1.0
Pleural Fluid alone Positive	17.2% (10/58)	10.3% (6/58) p = 0.03	3.4% (2/58) p = 0.50
Total positivity	70.6% (41/58) p<0.0001	22.4% (13/58) p = 0.0005	10.3% (6/58) p = 0.038

**NOTE**. AFB, acid fast bacilli; N-PCR, 116 bp amplicon generated by Nested-PCR assay specific for *Mycobacterium tuberculosis*; Culture, AFB Growth detected on LJ, 7H9 and/MGIT.

### Comparison of N-PCR assay results of AFB positive Vs AFB Negative pleural fluid samples

Ten of the 16 (62.5%) and 20 of the 42 (47.6%) pleural fluid samples were positive by the N-PCR assay derived from confirmed & probable tuberculous pleural effusion patients (Table 4). Inclusion of sputum samples enhanced the number of positive cases in both category of patients. In case of confirmed AFB positive cases of pleural TB, the detection of *Mtb* by N-PCR increased from 10 to 14 patients. Similarly in case of AFB negative cases but clinically diagnosed as pleural TB, the detection of *Mtb* increased from 20 to 27 patients. Inclusion of sputum enhanced the sensitivity of *Mtb* detection by N-PCR assay in confirmed (62.5 to 87.5%) and as well as in probable but AFB negative cases (47.6 to 64.2%) of pleural TB. By the N-PCR assay distinction between confirmed and clinically suspected cases of pleural TB was not feasible.

**Table 4:** Comparison of N-PCR assay results of samples derived from 58 clinically diagnosed tuberculous pleural effusion patients: 16 AFB positive Vs 42 AFB Negative samples.

S.No	Sample positive	Results of N-PCR in samples derived from tuberculous pleural effusion patients		Sensitivity (%)	PPV (%)	NPV (%)	OR	CI	P value
		Confirmed Tuberculous* (16)	Probable Tuberculous**(42)						
1	Pleural Fluid alone	2	8	12.5	20.0	70.8	0.60	0.11–3.2	ns
2	Sputum alone	4	7	25.0	36.3	74.4	1.66	0.41–6.71	ns
3	Both positive	8	12	50.0	40.0	78.9	2.5	0.76–8.1	ns
4	Any one/both	14	27	87.5	34.1	88.2	3.88	0.7–19.4	ns

**NOTE**. PPV, positive predictive value; NPV, negative predictive value; OR, odds ratio; CI, confidence interval.

* Categorized based on detection of AFB by smear microscopy/isolation by culture.

** Categorized based on clinical criteria detailed in methods, but no AFB detected smear microscopy/isolation by culture.

ns  =  not significant.

### Correlation of Direct detection of Mycobacteria in clinical samples by N-PCR assay Vs biochemical identification of isolates

The results of the N-PCR assay, AFB smear microscopy, isolation by culture and biochemical identification of the isolates has been detailed in Table 5. In all 13 isolates were obtained from 58 pleural tuberculosis patients. Six isolates were from pleural fluid (SPF-8,24,48,59A,101,103; Table 5) and additional seven isolates were obtained from the culture of the sputum (SPF-25,45,59,75A,78,80,85; Table 5) of these TPE patients. Of the 6 samples (2 pleural fluid and 4 sputum samples) which were smear AFB positive, isolates were obtained from 1 pleural fluid (SPF101) and 2 sputum samples (SPF-80, 85; Table 5) respectively. Direct detection and identification of mycobacteria in the sample was done by the N-PCR in 2 (SPF 85,101, Table 5) of these samples. The third sample (SPF 80) which was AFB smear and culture positive was N-PCR negative. This AFB isolate was characterized as *M.chelonae*, hence was not detected by the N-PCR assay, which is specific for detection of *Mtb* and *Mbo*. Sample SPF 8 (Table 5) was smear AFB negative but culture AFB positive. The isolate was N-PCR negative. This isolate was identified as *M.fortuitum*. No mycobacteria were isolated or detected in Non-TPE (Controls). Comparing the direct identification of *Mtb* by the N-PCR assay, a complete concordance was evident in the 13 culture positive samples, (Table 5). In eleven samples Mycobacterial isolates were identified by biochemical criteria as *Mtb*. In all theses samples the presence of *Mtb* was directly detected by the N-PCR assay prior to its isolation and identification.

**Table 5:** Correlation of direct detection of Mycobacteria in confirmed tuberculous pleural effusion samples by N-PCR assay Vs Detection of AFB by microscopy, isolation by culture and biochemical identification of the isolates.

S.No	Sample Code	Source	N-PCR	AFB	Culture	Biochemical Characterization
1	SPF-8	Pleural fluid	–	–	+	*M. fortuitum*
		Sputum	–	–	–	
2	SPF-24	Pleural fluid	*Mtb*	–	+	*Mtb*
		Sputum	*Mtb*	–		
3	SPF-48	Pleural Fluid	*Mtb*	–	+	*Mtb*
		Sputum	*Mtb*	–	–	
4	SPF-59A	Pleural Fluid	*Mtb*	–	+	*Mtb*
		Sputum	*Mtb*	–	–	
5	SPF-101	Pleural Fluid	*Mtb*	+	+	*Mtb*
		Sputum	–	–	–	
6	SPF-103	Pleural Fluid	*Mtb*	–	+	*Mtb*
		Sputum	*–*	–	–	
7	SPF-25	Pleural fluid	*–*	–	–	*Mtb*
		Sputum	*Mtb*	–	+	
8	SPF-45	Pleural fluid	*–*	–	–	*Mtb*
		Sputum	*Mtb*	–	+	
9	SPF-59	Pleural fluid	*–*	–	–	*Mtb*
		Sputum	*Mtb*	–	+	
10	SPF-75A	Pleural fluid	*Mtb*	–	–	*Mtb*
		Sputum	*Mtb*	–	+	
11	SPF-78	Pleural fluid	*Mtb*	–	–	*Mtb*
		Sputum	*Mtb*	–	+	
12	SPF-80	Pleural fluid	–	–	–	*M. Chelonae*
		Sputum	–	+	+	
13	SPF-85	Pleural fluid	*–*	–	–	*Mtb*
		Sputum	*Mtb*	+	+	
14	SPF-62	Pleural fluid	*Mtb*	+	–	–
		Sputum	*Mtb*	–	–	
15	SPF-34	Pleural fluid	*Mtb*	–	–	–
		Sputum	*Mtb*	+	–	
16	SPF-18	Pleural fluid	*Mtb*	–	–	–
		Sputum	–	+	–	

**NOTE**. AFB, acid fast bacilli; N-PCR, 116 bp amplicon generated by Nested-PCR assay specific for *Mycobacterium tuberculosis*; Culture, AFB Growth detected on LJ, 7H9 and/MGIT; Biochemical characterization of the isolates, detailed in methods.

## Discussion

Mycobacterial load is limiting in samples of pleural tuberculosis, hence its isolation and identification is demanding [16], [5]. Direct examination of pleural fluids by Ziehl-Neelsen staining requires bacillary densities of 10,000/ml, whereas for isolation by culture 10-100 viable bacilli are needed [17]. Detection of mycobacteria in suspected cases of pleural effusion has been shown to be augmented by the inclusion of sputum. Variable sensitivity ranging from 3.5 to 100% of isolation by culture from sputum collected from tuberculous pleural effusion patients has been reported, [18]–[24]. However in general the isolation of *Mtb* from pleural fluid (1.7 to 24.5%) has been lower compared to sputum, [18]–[20], [22]–[23], [25], with the exception of the reports by Seibert *et al* & Epstein *et al* [21], [24]. Similarly the direct AFB smear microscopy of pleural fluid samples derived from pleural effusion TB patients was lower (0–20%) as compared to sputum collected from these patients, [22], [25]–[27]. AFB positivity of sputum smears of pleural effusion patients ranged from 1.7–62.5% [19], [22]–[23], [25].

Besides inclusion of an assortment of samples from an individual patient, attempts have been made to use rapid reliable DNA amplification techniques for efficient diagnosis of tuberculosis [14], [23]. The sensitivity of the different PCR assay has been reported to be ranged from 43.4–73.8% [19], [26]–[29].

In the present study, we have evaluated the (1) inclusion of sputum along with pleural fluid and (2) the usefulness of the direct identification and detection of *Mtb* using an in house N-PCR assay. It can be seen that the detection of mycobacteria in pleural fluid and sputum samples varied. Ten pleural fluid samples were positive, whereas the sputum samples of these patients collected in tandem were negative. Similarly eleven sputum samples were positive whereas the pleural fluid samples of these patients were negative by the N-PCR assay. The inclusion of sputum samples of clinically diagnosed pleural TB patients increased the total number of pleural TB patients detected from 30 to 41. The sensitivity of the assay increased from 51.7 to 70.6%, (Table 1 & 3).

Similarly improvement in AFB microscopy, isolation and detection of mycobacteria was observed on inclusion of sputum along with pleural fluid for investigation. Inclusion of sputum enhanced the sensitivity of AFB smear microscopy from 3.4 to 10.3% and culture sensitivity enhanced from 10.3 to 22.4%, (Table 1 & 3). The sensitivity of these two classical techniques in combination with mycobacterial DNA detection techniques would substantially improve the diagnosis of pleural TB.

Of the 13 isolates obtained in the study, 11 were identified as *Mtb*. The remaining two isolates were characterized as *Mycobacterium fortuitum* and *Mycobacterium chelonae* respectively, (Table 5). There have been reports of these fast growing mycobacteria being isolated from cases of tuberculous pleurisy and empyema [30], [31]. Use of solid & liquid culture media in 69 patients samples (Pleural fluid & sputum) enhanced sensitivity from 5.8% (4/69) when using either solid/liquid media alone to 11.6% (8/69) when both solid as well as liquid media were used. On using automated system (MGIT) the sensitivity observed was 16.1% (5/31), which was higher than the combined efficiency of LJ and 7H9 media used in the study. Admittedly this sensitivity observed with the MGIT culture system would have improved with the inclusion of a solid media such as LJ media, which was not done.

The results of the N-PCR assay was correlated with clinical categorization of the patients, AFB smear microscopy and isolation of *Mtb* by culture. By the 3 techniques used in the study namely AFB smear microscopy, culture for mycobacteria and the specific N-PCR assay for *Mtb* and *Mbo*, mycobacteria were detected in samples exclusively derived from patients clinically diagnosed as patients of TPE. Comparing the specificity and sensitivity of the 3 techniques it was found that the N-PCR assay had the highest sensitivity (51.7 to 53.4%) compared to the remaining two techniques. None of the non-TB pleural fluid samples were positive by the N-PCR assay, (Table 1 & 2).

Estimation of adenosine deaminase (ADA) and IFN-γ in pleural fluid has been used widely as biochemical markers in the diagnosis of TB pleural effusion [26], [32]–[33], [5]. However these biomarkers are indicative of an inflammatory process in the pleural cavity and do not define or identify the aetiological agent. Besides, ADA is raised in pleural malignancies, lymphoproliferative and rheumatoid diseases [34].

The diagnosis of TPE has been considered to be definitive with the demonstration of *Mtb* in the examined sample. However the extended time periods required for isolating and identifying *Mtb* from samples of pleural effusion and the limited sensitivity of the techniques employed have hampered the diagnosis of TPE. Further, histolopathological examination of pleural biopsies for granulomatous inflammation consistent with tuberculosis has been reported to be more sensitive than pleural fluid culture [18], [26], [20]. Pleural fluid has been reported to be a poor source of mycobacteria [19]. Several investigators have reported that pleural biopsies to be the most reliable for diagnosis of TPE provided the appropriate site is available for examination [35], [25]. However, the invasiveness of the procedure, inability to obtain characteristic pleural tissue and the accompanied complications has been the limitations in this regard. In the present study irrespective of the technique used qualitative improvement in the detection of mycobacteria in TPE derived samples, was seen with the inclusion of sputum samples.

In conclusion, detection of Mycobacteria either by microscopy/classical microbiological techniques or by DNA amplification techniques in pleural effusion patients was enhanced by examining pleural fluid as well as sputum collected in tandem. Further comparing the three methods, *Mycobacterium tuberculosis* was detected by the N-PCR assay in 70.6% of the samples compared to 22.4% by culture and 10.3% by AFB smear microscopy. This improvement by molecular methods in identification of pathogens can be sustained by targeting appropriate gene targets and adopting sensitive detection techniques.
